# War on Waste: Challenges and Experiences in COVID-19 Waste Management

**DOI:** 10.1017/dmp.2021.171

**Published:** 2021-06-07

**Authors:** Naveen R. Gowda, Vijaydeep Siddharth, Khalid Inquillabi, D. K. Sharma

**Affiliations:** 1 Department of Hospital Administration, All India Institute of Medical Sciences, New Delhi, India; 2 Medical Superintendent, All India Institute of Medical Sciences, New Delhi, India

**Keywords:** COVID-19 waste, biomedical waste, waste management, healthcare re-engineering

## Abstract

**Objective:**

Coronavirus disease 2019 (COVID-19) has posed formidable challenges, including overwhelming biomedical waste management. Guidelines have been rapidly changing along with the mounting pressure of waste generation.

**Methods:**

These challenges were managed by smart re-engineering of structure and processes for the desired outcomes. Dedicated staff, in personal protective equipment with appropriate training, were deployed to collect waste using dedicated trolleys. A dedicated route plan was drawn with a dedicated elevator meant for COVID-19. A new temporary holding area was created. Dedicated trucks with requisite labels were deployed to transport COVID-19 waste to a common biomedical waste treatment facility. Communication challenges were addressed through timely circulars, which were further reinforced and reiterated during various on-going training programs.

**Results:**

Before the onset of COVID-19 pandemic, the amount of biomedical waste generated was 1.93 kg/bed/day; currently, the quantity of COVID-19 biomedical waste generated is 7.76 kg/COVID bed/day. Daily COVID-19 waste generation data are maintained and uploaded in an android application monitored by Central Pollution Control Board, Government of India. To date, none of the workers handling COVID-19 waste has acquired health-care associated COVID-19 infection, which reflects on the soundness of the new system and the infection control practices in the institute.

**Conclusions:**

A responsive leadership harmonizing with a robust communication and training system has augmented timely re-engineering of structure and processes for better outcomes in the war on waste.

The coronavirus disease 2019 (COVID-19) pandemic has posed unprecedented challenges on all fronts of the healthcare delivery. Right from the basic understanding of the pathogenesis, to case diagnosis and management, every step has been an enigma for the medical fraternity to crack. This naturally translated to chaos in the health-care system, and health-care facilities found it nearly impossible to function the same way we had known them to. News of increasing deaths in China and Italy, among other nations, which were initially the first and hardest hit, roused a natural sense of fear even among health-care professionals. From knowing the right thing to be done to actually implementing it on the ground, every step has been a challenge and has pushed everyone and every aspect of health care to the hilt.

One of the vital components of any health-care facility is biomedical waste management and has been no exception to this chaos. The deluge of “Infodemic” saw all kinds of information doing rounds among general public and healthcare workers alike. The World Health Organization, many international agencies and nations have been grappling with this pandemic with new guidelines flowing in even before the previous ones could be implemented.^1–4^

COVID-19 has reportedly increased needless practices and wastage in health care,^5^ which translates into increased overall waste generation. In addition, if not properly managed, this medical waste can potentially harm health-care workers and other people and accelerate disease spread. Globally many approaches are being tried, such as the reverse logistics system at Wuhan^6^ among others.

Considering the acuity of the situation and specific needs for India, it was imperative to evolve our own approach toward COVID-19 waste management. The Central Pollution Control Board (CPCB) is a statutory body under the government of India and the nodal authority, which issues guidelines and directives to be followed by various State Pollution Control Boards (SPCBs) and committees for various aspects pertaining to pollution control, including biomedical waste management from time to time.^7^

The CPCB has issued comprehensive guidelines for COVID-19 waste management in isolation wards, sample collection centers and labs, and quarantine camps/home care, and for the duties of the Common Biomedical Waste Treatment Facility (CBWTF) and duties of SPCBs in March 2020.^8^ These guidelines evolved dynamically with four revisions starting from March 2020, based on the feedback from on-ground experiences of various stakeholders and experts, which aided in managing the COVID waste effectively.

## Problem Statement

**Figure 1.**
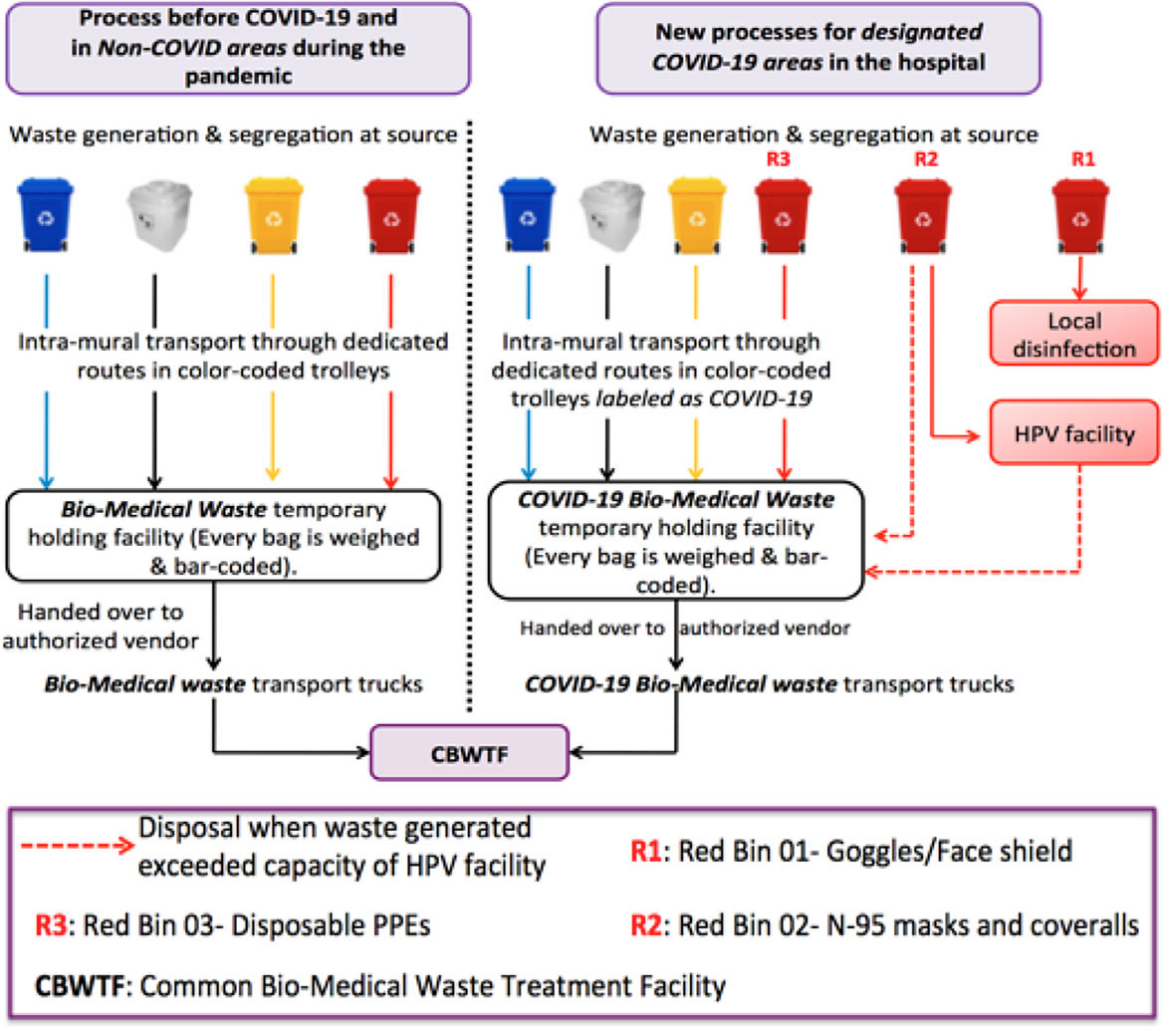
Prepandemic biomedical waste management process vis a vis process re-engineering for handling COVID 19 biomedical waste.

Before the beginning of the pandemic, biomedical waste was being segregated and stored in color-coded bins (having single liners) kept in the dirty utility room of the various patient care areas. From there, it is taken by the biomedical waste handlers to the temporary waste storage site and, thereafter, it is taken to the CBWTF for final disposal. However, management of COVID-19 waste has been a challenge, as it required a complete beginning-to-end rework of the system. In addition to the biomedical waste being stored in dirty utility rooms, biomedical waste is also being segregated and stored in doffing areas created across the hospital in various patient care areas catering to COVID 19 suspected/confirmed cases (Figure 1). Managing the large volume of COVID-19 biomedical waste in the midst of rapidly evolving guidelines and widespread paranoia, at the same time ensuring that the spread of COVID 19 infection is prevented within the hospital, has been a formidable challenge.

## Structural and Process Re-engineering

Right from the point of waste generation, through to collection, intra-mural transportation to temporary storage facilities, to handing it over to the CBWTF operator, the entire process of biomedical waste management had to be re-engineered (Figure 1).

At our institute, which is an Institute of National Importance providing tertiary care to admitted patients, some patient care areas were converted into COVID facilities while other areas continued to serve non-COVID patients. The pre-existing processes for biomedical waste management are being continued in non-COVID areas during the COVID-19 pandemic. Processes were modified and evolved to manage COVID-19 biomedical waste generated from designated COVID-19 areas, which entailed its own set of problems to begin with. Our challenges and experiences can be better understood through the time tested *Donabedian approach of structure, process, and outcome.* ^9^

Major *structural* revamps were a formidable challenge. Dedicated wards in the hospital were earmarked for COVID-19 suspects and patients, which helped to limit the points of COVID waste generation. In line with the CPCB guidelines, the colors of bins were not changed, and the waste collected was double bagged. At the institutional level, a system of 3 red bins was introduced (Table 1).

**Table 1.** Three red bin system for biomedical waste management in doffing areas of the patient care facilities catering to COVID 19 suspect/confirmed cases

Name of the red bin	Contaminated PPE	Management of waste
Red bin 01	Goggles/face shield	0.5% sodium hypochlorite freshly prepared solution/70% alcohol (Bacillol)
Red bin 02	N-95 masks and coveralls (white)	Store in double bags (red) and hand over to the authorized Hospital Attendant/Sanitary Attendant who is deputized from the sanitation and housekeeping services for collection from each patient care area; collection of these PPEs will be done twice daily
Red bin 03	Disposable PPEs	To be handed over to authorized waste collecting staff of M/s Biotic Waste Solutions Pvt. Ltd.

Abbreviation: PPE, personal protective equipment.

One red bin was meant for only the biosafety coveralls, which were sent to the hydrogen peroxide vaporization (HPV) facility for sterilization and re-use; another red bin was for collection and on-site disinfection of goggles and face-shields with sodium hypochlorite solution and the third bin was continued for contaminated disposable single use plastic items as per biomedical waste management rules. In view of projected surge in cases, it became imminent to be prepared for recycling personal protective equipment (PPE); therefore, red bin 02 was dedicated for collecting coveralls that were then sent to the HPV facility for sterilization and eventual re-use.^10^

The next challenge was waste collection. Dedicated staff in PPE with appropriate training were deployed to collect waste using dedicated trolleys. With widespread paranoia and fear, transporting a trolley labeled as COVID-19 within the hospital was fraught with challenges. A dedicated route plan was drawn with a dedicated elevator meant for COVID-19. A new temporary holding area was created, separate from the common biomedical waste collection point. The paucity of space was a major challenge as unidirectional traffic flow had to be established to minimize crisscrossing of paths with movement of people, staff, and hospital supplies. Procurement of biomedical waste collection bins and additional trolleys was done expeditiously to cater to increased demand owing to segregation and creation of new areas.

COVID-19 waste was handed over to the authorized services agency twice a day; however, the processing of waste at the CBWTF was not immediate because the CBWTF plant was overwhelmed owing to increased waste generation. This inundation was probably due to the increased use of disposables and general waste being contaminated and treated as biomedical waste. COVID-19 waste posed enormous challenges to the entire system of biomedical waste collection, which had a cascading effect on other health-care facilities as well.^11^

At the beginning of the pandemic, in some health-care facilities, all COVID waste was being sent for incineration, a process that was corrected with increasing awareness. Periodic revisions of the CPCB guidelines have tried to address and correct the practice of discarding general waste from COVID-19 suspected patients and confirmed patients to yellow bags. Arrangements were also made to transport COVID-19 waste in dedicated trucks with requisite labels to CBWTF.

Various *processes* had to be changed. Every modification required new learning and behavioral change on the part of employees. Therefore, people are at the center of all processes. In the hour of crisis, owing to lockdown and logistic limitations, some employees could not turn up for work; however, available manpower was redistributed as needed.

The institute’s long-term investment on creating dedicated teams of infection control nurses (ICNs), nurses for continued nursing education (CNEs), and nursing informatics specialists (NIS) paid rich dividends. They have always been involved in training and awareness activities and continued the stride at the time of need. Our communication channels have stood the test of time due to pre-established systems involving ICNs and CNEs. A pre-set culture of continuous education and training came in handy when we had to train and re-train all cadres of staff with rapidly changing guidelines.

All new changes in COVID-19 waste and biomedical waste management were disseminated to all employees across the institute through informative circulars, which were further reinforced and reiterated during various on-going training programs. Regular training is being conducted for all cadres of staff and Trainer of Trainers (ToT) is being conducted to increase outreach. These efforts have improved dissemination of information and thus ensured translation to results on ground.

The overall *outcomes* have been very promising and reassuring thus far. All of the systems and processes that were put in place have resulted in the effective management of COVID waste. There has been a phenomenal jump in the amount of biomedical waste generated from COVID facilities, and the hospital system has been geared up to handle this surge; however, infrastructure at the CBWTF level needs strengthening. All biomedical waste generated is weighed before dispatching to the CBWTF, and data of daily biomedical waste generation is being maintained.

## Discussion

Before the onset of the COVID-19 pandemic, biomedical waste (excluding general waste) generated was 1.93 kg/bed/day. After onset of COVID-19, there has been a phenomenal jump in the amount of COVID-19 biomedical waste generated from designated COVID-19 areas to 7.76 kg/COVID bed/day. Due to lockdown, elective admissions were stopped in non-COVID wards;therefore, waste generation from COVID-designated areas has been recorded.

**Figure 2.**
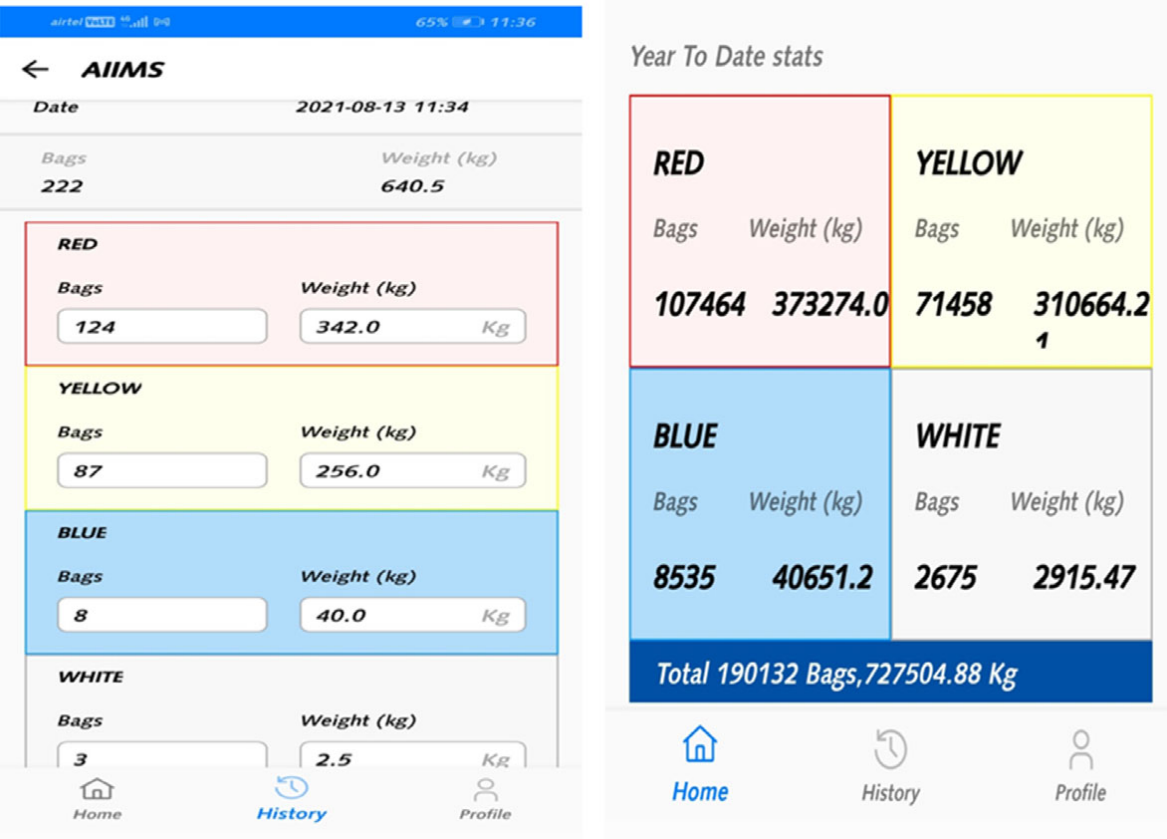
The CPCB COVID-19 BWM app for daily data uploading of COVID 19 biomedical waste data.

All contaminated waste, including general waste, from designated COVID areas, was considered as biomedical waste, per the CPCB guidelines. Data with regard to COVID waste generation are submitted on a daily basis to CPCB through an Android-based app COVID19BWM^12^ (Figure 2). This is in addition to the biomedical waste generated from other non-COVID areas of the hospital. The structural and process re-engineering were vital to manage the surge in the amount of COVID waste, which was 4-fold more than bio-medical waste generated per bed before the onset of COVID-19.

Right from the stage of waste generation and segregation through intra-mural transport, temporary storage, and finally dispatch to CBWTF, every step required changes in practices (Figure 1), which were successfully implemented. Thus, the process-oriented approach of re-thinking and re-working every step to minimize spread of infection has been helpful during the pandemic, which has also been reiterated in other studies from China.^13^

There are 40 helpers involved in directly collecting and handling COVID waste and 5 supervisors who oversaw the overall waste collection activities. To date, none of them have acquired health-care associated COVID infection, which reflects on the soundness of the new system and the infection control practices in the institute. The feedback loop right from the ground level up to CPCB has helped to continuously and timely evolve the waste management practices.

## Conclusions

Regulatory changes were made in biomedical waste management, for facilities catering to COVID-19 suspected cases/confirmed cases, which required process modifications for handling of COVID-19 biomedical waste. These changes evolved as the pandemic progressed, posing challenges for health-care facilities with the amount of waste being generated being the intricate issue to manage. A responsive leadership harmonizing with a robust communication and training system has helped obtain timely changes in structure and processes for better outcomes. Thus, the war on waste seems to have been won, and certainly has not been lost.

## References

[1] Ministry of Health and Family Welfare, Government of India. National Training of Trainers for COVID-19: Environmental cleaning, disinfection and bio-medical waste management. https://health.odisha.gov.in/pdf/CTR/BCTD/Environmental-cleaning-disinfection-bio-medical-waste-management.pdf. Accessed June 8, 2021.

[2] National Center for Immunization and Respiratory Diseases, Division of Viral Diseases. Interim infection prevention and control recommendations for patients with suspected or confirmed coronavirus disease 2019 (COVID-19) in healthcare settings. CDC. 2020;2:1-10. https://www.cdc.gov/coronavirus/2019-ncov/infection-control/control-recommendations.html.

[3] UNICEF. COVID-19 emergency preparedness and response WASH and IPC guidlines. 2020. https://www.unicef.org/media/66386/file/WASH-COVID-19-infection-prevention-and-control-in-health-care-facilities-2020.pdf. Accessed June 8, 2021.

[4] Bodine SP. United States Environmental Protection Agency. Proc Water Environ Fed. 2012;2005(16):726-737. doi: 10.2175/193864705783867675

[5] Warner MA. Stop doing needless things! Saving healthcare resources during COVID-19 and beyond. J Gen Intern Med. 2020;35(7):2186-2188. doi: 10.1007/s11606-020-05863-6 32383149 PMC7205372

[6] Yu H, Sun X, Solvang WD, et al. Reverse logistics network design for effective management of medical waste in epidemic outbreaks: insights from the coronavirus disease 2019 (COVID-19) in Wuhan (China). Int J Environ Res Public Health. 2020;17(5):1770. 10.3390/ijerph17051770 PMC7084373 32182811

[7] Central Pollution Control Board. Official website. https://cpcb.nic.in. Accessed June 8, 2021.

[8] Central Pollution Control Board. Guidelines for handling, treatment, and disposal of waste generated during treatment/diagnosis/quarantine of COVID-19 patients. 2020. http://www.uppcb.com/pdf/Guidelines_190320.pdf. Accessed June 8, 2021.

[9] Rublee DA. The quality of care: how can it be assessed? JAMA. 1989;261(8):1151. doi: 10.1001/jama.1989.03420080065026 2915435

[10] Kumar P, Killedar M, Singh G. Adaptation of the ‘assembly line’ and ‘brick system’ techniques for hospital resource management of personal protective equipment, as preparedness for mitigating the impact of the COVID-19 pandemic in a large public hospital in India. J Hosp Infect. 105(4):787-789. 32450194 10.1016/j.jhin.2020.05.029 PMC7242941

[11] Mohan V. Dealing with biomedical waste in the time of Covid-19 presents huge challenge. *The Times of India*. https://timesofindia.indiatimes.com/india/dealing-with-biomedical-waste-in-the-time-of-covid-19-presents-huge-challenge/articleshow/75905790.cms. Accessed June 8, 2021.

[12] Google Play. https://play.google.com/store/apps/details?id=com.cpcb.bmw. Accessed August 29, 2020.

[13] Peng J, Wu X, Wang R, et al. Medical waste management practice during the 2019-2020 novel coronavirus pandemic: experience in a general hospital. Am J Infect Control. 2020;48(8):918-921. 32504761 10.1016/j.ajic.2020.05.035 PMC7267792

